# Effect of Algal Lectin Siye on Proliferation and Apoptosis of Breast and Colon Cancer Cells

**DOI:** 10.3390/md24060199

**Published:** 2026-06-04

**Authors:** Xiaobo Zhang, Jianfei Ma, Jiahao Ma, Tongli Xu, Xianfeng Ruan, Mengyu Pang, Tian Wang, Lu Wang

**Affiliations:** 1 School of Pharmacy, Yantai University, Yantai 264005, China; sysm_l727@163.com (X.Z.); bluewangtian@hotmail.com (T.W.); 2 State Key Laboratory of Medicinal Chemical Biology, Nankai University, Tianjin 300071, China; 3 College of Life Science, Nankai University, Tianjin 300071, China; 4 BayVax Biotech Limited, 17 Science Park West Avenue, Hong Kong Science Park, Shatin, Hong Kong SAR, China; 5 School of Biomedical Sciences, Li Ka Shing Faculty of Medicine, The University of Hong Kong, 21 Sassoon Road, Pokfulam, Hong Kong SAR, China; 6 Materials Innovation Institute for Life Sciences and Energy, The University of Hong Kong, 1 Honghua Road, Shenzhen 518016, China; 7 Key Laboratory of Coastal Biology and Biological Resource Utilization, Yantai Institute of Coastal Zone Research, Chinese Academy of Sciences, Yantai 264003, China

**Keywords:** red alga, lectin, anti-tumor, apoptosis, colon cancer, breast cancer, artificial intelligence, genome mining, marine drug discovery

## Abstract

Lectins are carbohydrate-binding proteins, some of which exhibit significant anti-tumor activity. Siye is a lectin derived from the red alga *Kappaphycus alvarezii* that was previously discovered using an artificial intelligence-guided genome mining strategy and shown to exert cytotoxic effects against several human cancer cell lines, including breast adenocarcinoma HCC1937. Based on the presence of shared glycopatterns between breast and colon cancers, we hypothesized that Siye may also exhibit anti-tumor activity against colon cancer cells. The cytotoxic effect of Siye on human colon cancer HCT116 cells was evaluated using the CCK-8 assay. Apoptosis was assessed by flow cytometry with Annexin V-FITC/PI staining. Expression levels of apoptosis-related genes (*Bax*, *Bcl-2*, *Casp3*, *Casp8*, *Casp9*, and *TP53*) were determined by qRT-PCR. Competitive inhibition assays using mannan were performed to assess the role of cell surface glycan binding. Siye significantly reduced the viability of HCT116 cells in a dose-dependent manner, with an IC_50_ value of 14.065 μg/mL (=0.488 μM). Flow cytometry revealed that Siye promoted both early and late apoptosis in HCT116 cells, whereas in HCC1937 cells, the effect was primarily on early apoptosis. Mechanistically, Siye significantly upregulated the expression of the pro-apoptotic genes *Bax* (*p* < 0.05) and *Casp9* (*p* < 0.001) in HCT116 cells, while in HCC1937 cells, *Casp9* expression was significantly increased (*p* < 0.001). Morphological changes, including cell rounding and agglutination, were observed within 4 h of Siye treatment in both cell lines and were attenuated by co-treatment with mannan, suggesting that Siye-induced morphological changes are associated with binding to cell surface glycans. This study suggests that the red algal lectin Siye exerts anti-tumor effects against colon cancer HCT116 cells by inducing caspase-associated apoptosis. The differential apoptotic response between HCC1937 and HCT116 cells suggests cell-type-specific mechanisms. These findings extend the known anti-tumor activity spectrum of AI-discovered red algal lectin Siye and provide a basis for further investigation of its glycan-associated cellular effects and marine drug discovery potential.

## 1. Introduction

Lectins are a class of proteins that bind specifically to carbohydrates, and have attracted considerable interest as potential therapeutic agents due to their anti-tumor [1,2,3], anti-viral [4,5,6], and anti-inflammatory properties [7,8]. Although lectins are widely distributed across plants, animals, fungi, bacteria, and viruses, algal lectins remain relatively underexplored despite the high biodiversity of algae [9,10].

Among marine-derived lectins, the *Oscillatoria agardhii* agglutinin homolog (OAAH) family has shown promising anticancer potential [9,11], including low molecular weight, independence from divalent cations for activity, high specificity for complex oligosaccharides, and negligible affinity for monosaccharides [12,13].

Our group previously identified a novel OAAH family lectin, named Siye, from the genome of the red alga *Kappaphycus alvarezii*, using a genome mining method guided by evolutionary theory and artificial intelligence (AI), and demonstrated its cytotoxicity against several human cancer cell lines (IC_50_ values ranging from 0.11 to 3.95 μg/mL, i.e., 0.0039–0.1400 μM), including breast adenocarcinoma HCC1937, lung carcinoma A549, liver cancer HepG2, and promyelocytic leukemia HL60 [14]. This AI-assisted discovery strategy provided a rational route for identifying bioactive lectins from marine genomic resources. In recent years, artificial intelligence (AI) has emerged as a powerful tool in marine drug discovery, enabling the rapid identification of bioactive compounds from large-scale genomic datasets [15,16]. AI-assisted approaches, including machine learning-based sequence screening, structural prediction, and functional annotation, have significantly improved the efficiency and accuracy of discovering novel marine-derived biomolecules, particularly for structurally complex proteins such as lectins [17,18,19].

Notably, common glycopatterns have been reported in the serum of patients with breast, lung, and colorectal cancers [20], many of which are included in the glycan target set of Siye. This observation raises the possibility that Siye may also exert anti-tumor effects against colon cancer cells. In the present study, we investigated the cytotoxic and pro-apoptotic effects of Siye on human colon cancer HCT116 cells and compared its mechanism of action with that in breast cancer HCC1937 cells. This work contributes to a deeper understanding of the broad-spectrum anti-tumor activity and common mechanisms of OAAH family lectins. Furthermore, this study highlights the potential of AI-assisted genome mining as an effective strategy to bridge computational prediction and experimental validation in marine drug discovery [15].

## 2. Results

### 2.1. Effect of Siye on Tumor Cell Viability

**Figure 1:**
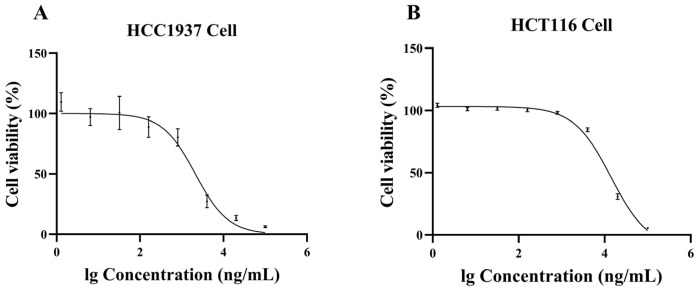
Cytotoxic effects of Siye on tumor cells. Cells were treated with serial dilutions of Siye (100 μg/mL to 0.00128 μg/mL) for 24 h. (**A**) HCC1937 human breast cancer cells, IC_50_ = 2.112 μg/mL (=0.0733 μM, data from three independent experiments, previously reported [14]); (**B**) HCT116 human colon cancer cells, IC_50_ = 14.065 μg/mL (=0.488 μM, data from three independent experiments). Data are presented as mean ± SEM.

To evaluate the cytotoxic effects of Siye, cancer cells were treated with increasing concentrations of the lectin for 24 h. As shown in Figure 1, Siye induced a dose-dependent reduction in cell viability in both breast cancer HCC1937 cells (Figure 1A) and colon cancer HCT116 cells (Figure 1B). The IC_50_ values were calculated as 2.112 μg/mL (=0.0733 μM; 95% CI: 1.357–3.326 μg/mL) for HCC1937 (consistent with our previous report [14]) and 14.065 μg/mL (=0.488 μM; 95% CI: 12.092–16.377 μg/mL) for HCT116. These results indicate that Siye exhibits dose-dependent cytotoxicity in HCT116 cells under the present experimental conditions.

### 2.2. Glycan Competitive Inhibition of Siye-Induced Morphological Changes

**Figure 2:**
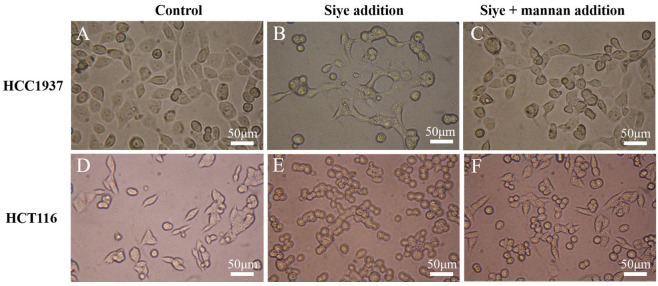
Morphological changes in tumor cells after treatment with Siye alone or Siye + mannan. Cells were treated with Siye alone or Siye pre-incubated with mannan for 4 h. Equal volumes of treatment solutions were added across groups. (**A**–**C**) HCC1937 cells: control (**A**), Siye-treated (**B**), Siye + mannan (**C**); (**D**–**F**) HCT116 cells: control (**D**), Siye-treated (**E**), Siye + mannan (**F**).

To determine whether the cellular effects of Siye are mediated by binding to cell surface glycans, HCC1937 and HCT116 cells were treated with Siye alone or in combination with mannan, a competitive inhibitor. Morphological changes were monitored every hour, with notable alterations observed at 4 h post-treatment, while HCC1937 cells (Figure 2B) and HCT116 cells became rounded and agglutinated (Figure 2E). Co-treatment with mannan markedly attenuated these morphological changes in both cell lines (Figure 2C,F), suggesting that Siye-induced morphological changes are associated with binding to cell surface carbohydrates.

### 2.3. Effect of Siye on Tumor Cell Apoptosis

**Figure 3:**
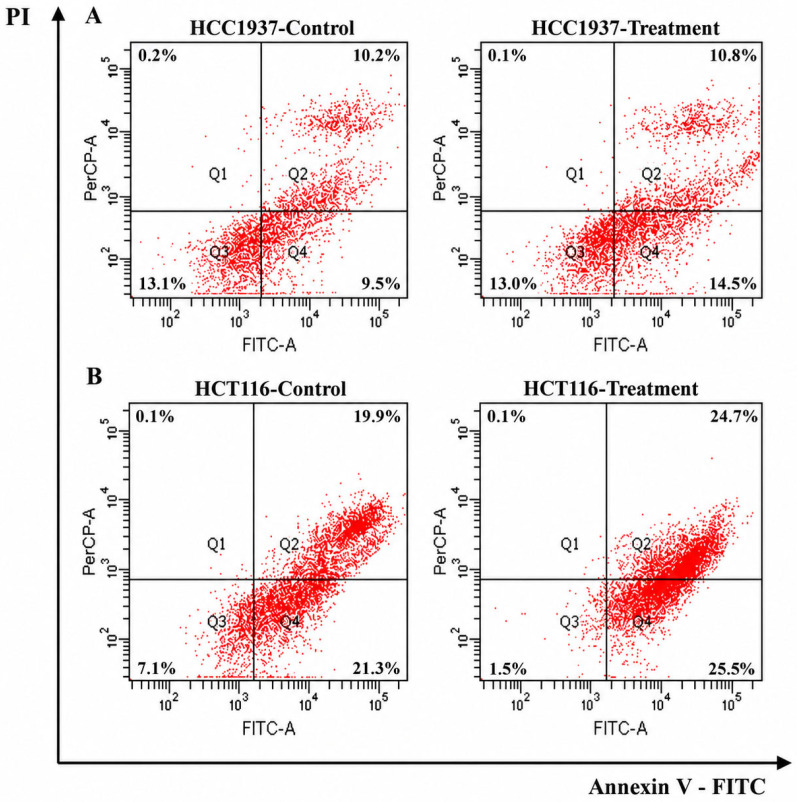
Flow cytometry analysis of apoptosis induced by Siye. Cells were treated with Siye at IC_50_ concentrations for 24 h and stained with Annexin V-FITC/PI. Q1: necrotic cells; Q2: late apoptotic cells; Q3: viable cells; Q4: early apoptotic cells. (**A**) HCC1937 cells; (**B**) HCT116 cells.

Flow cytometry analysis using Annexin V-FITC/PI staining was performed to assess Siye-induced apoptosis after 24 h of treatment at IC_50_ concentrations. In HCC1937 cells (Figure 3A), the early apoptosis rate increased from 9.5% in the control group to 14.5% in the Siye-treated group, whereas late apoptosis showed a modest increase from 10.2% to 10.8%. In HCT116 cells (Figure 3B), Siye treatment increased early apoptosis from 21.3% to 25.5% and late apoptosis from 19.9% to 24.7%. These results indicate that Siye treatment at the IC_50_ concentration promotes apoptosis in both HCC1937 and HCT116 cells, with HCC1937 cells showing a predominant increase in early apoptosis and HCT116 cells showing increases in both early and late apoptosis.

### 2.4. Effect of Siye on Apoptosis-Related Gene Expression

**Figure 4:**
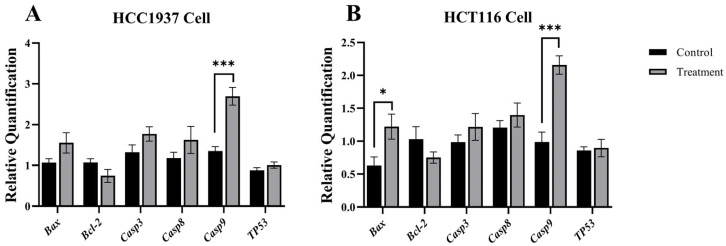
Expression of apoptosis-related genes after Siye treatment. Gene expression levels were measured by qRT-PCR using the 2^−ΔΔCT^ method, with *GAPDH* as an internal control. Grey bars: Siye-treated cells; black bars: control cells. (**A**) HCC1937 cells; (**B**) HCT116 cells. Data are presented as mean ± SEM from three independent experiments. * *p* < 0.05, *** *p* < 0.001.

To further explore apoptosis-related gene expression changes after Siye treatment, we examined the expression of key apoptosis-related genes (*Bax*, *Bcl-2*, *Casp3*, *Casp8*, *Casp9*, and *TP53*) by qRT-PCR after 24 h of Siye treatment at IC_50_ concentrations (Figure 4). In HCC1937 cells (Figure 4A), expression of the pro-apoptotic gene *Casp9* was significantly upregulated (*p* < 0.001). In HCT116 cells (Figure 4B), significant upregulation was observed for both *Bax* (*p* < 0.05) and *Casp9* (*p* < 0.001). The *Bcl-2*/*Bax* ratio decreased in both cell lines following Siye treatment. These findings suggest that Siye-induced apoptosis may be associated with changes in apoptosis-related gene expression, particularly *Casp9* in both cell lines and *Bax* in HCT116 cells.

## 3. Discussion

### 3.1. Siye Exhibits Broad-Spectrum Anti-Tumor Activity with Cell-Type Specificity

In our previous study, the OAAH family lectin Siye, identified from the genome of the red alga *Kappaphycus alvarezii*, was shown to exert significant cytotoxicity against several human cancer cell lines, including HCC1937, HepG2, A549, and HL60, with IC_50_ values ranging from 0.11 to 3.95 μg/mL (i.e., 0.0039–0.1400 μM) [14]. The previous study suggested that the anti-tumor effect of Siye may be associated with caspase- and p53-related apoptotic pathways [14]. In the present study, we extended these findings by demonstrating that Siye also exhibits potent cytotoxic effects on the colon cancer HCT116 cells, with IC_50_ values of 14.065 μg/mL (=0.488 μM). These results further support the broad-spectrum anti-tumor potential of Siye.

Notably, the IC_50_ values varied considerably across different cancer cell lines, with HCT116 being less sensitive than HCC1937, HepG2, and A549. This differential sensitivity may reflect variations in the surface glycan profiles among cancer types. Lectin Siye exhibits high binding affinity for mannose-containing glycans [14]. Previous studies have shown that high-mannose-type N-glycans are altered in breast cancer cells and tissues [21] and that high-mannose N-glycans are present on the surface of colorectal cancer cell lines [22]. Moreover, N-glycosylation patterns vary across cancer types and cellular contexts [23]. Therefore, the different cellular responses observed between HCC1937 and HCT116 cells may be partly related to differences in the abundance or accessibility of Siye-recognized glycan structures on the cell surface.

### 3.2. Siye May Induce Apoptosis Through Caspase-Associated Pathways

Flow cytometry analysis revealed that Siye treatment at the IC_50_ concentration promoted early apoptosis in HCC1937 cells, whereas in HCT116 cells, both early and late apoptosis were significantly increased. This discrepancy suggests that the apoptotic response to Siye may be executed more efficiently in HCT116 cells, possibly due to the differential activation of downstream apoptotic effectors. Consistently, qRT-PCR analysis showed that Siye significantly upregulated the pro-apoptotic genes *Casp9* in both cell lines and *Bax* in HCT116 cells. The increased *Bax*/*Casp9* mRNA expression, together with a decreased *Bcl-2*/*Bax* ratio, suggests that Siye-induced apoptosis may be associated with the intrinsic mitochondrial apoptotic pathway. However, because the current evidence is based mainly on transcriptional analysis, protein-level and functional validation, particularly the detection of cleaved caspases and cleaved PARP, is required before confirming the activation of a specific apoptotic pathway.

Interestingly, while *Casp9* was significantly upregulated in both cell lines, *Bax* upregulation reached statistical significance only in HCT116 cells. This may reflect differences in the transcriptional regulation of *Bax* between the two cell lines, or the involvement of alternative pro-apoptotic mediators in HCC1937 cells. Notably, HCC1937 harbors a *TP53* mutation [24], which may impair the p53-dependent transcriptional activation of *Bax* [25]. In contrast, HCT116 retains wild-type p53 [26], allowing for a more robust *Bax*-mediated apoptotic response [27]. These findings highlight the importance of considering the genetic background of cancer cells when evaluating lectin-induced apoptosis.

### 3.3. Cell Surface Glycan Binding Is Critical for Siye-Induced Agglutination and Morphological Changes

In line with our previous observations in A549 and HepG2 cells [14], Siye induced significant morphological changes and agglutination in both HCC1937 and HCT116 cells within 4 h of treatment. These changes were attenuated by co-treatment with mannan, a competitive inhibitor commonly used for mannose-binding lectins. These results suggest that Siye exerts its initial cellular effects primarily through binding to mannose-containing glycans on the cancer cell surface. Such lectin–glycan interactions may trigger downstream signaling cascades leading to apoptosis, although the precise link between agglutination and apoptotic signaling remains to be elucidated.

### 3.4. Comparison with Previously Reported OAAH Family Lectins

The anti-tumor activity of Siye is consistent with that of other OAAH family lectins, such as ESA from *Eucheuma serra* [28], SfL from *Solieria filiformis* [29], and KSL from *Kappaphycus striatus* [30]. Structurally, Siye shares the characteristic four-tandem-repeat β-barrel fold common to many red algal OAAH lectins. However, Siye exhibits superior potency, with IC_50_ values of 0.1085 μg/mL (=0.0039 μM) against HepG2 cells and 0.7786 μg/mL (=0.0276 μM) against A549 cells (previously reported [14]), which are substantially lower than the reported IC_50_ values of KSL (0.80–1.94 μM against various cancer cell lines) [30].

Consistently, in the present work, Siye was shown to have IC_50_ values of 2.112 μg/mL (=0.0733 μM) against HCC1937 human breast cancer cells and 14.065 μg/mL (=0.488 μM) against HCT116 human colon cancer cells. In comparison, SfL required 125 μg/mL (=4.5 μM) to achieve 50% viability reduction in MCF-7 human breast cancer cells [29], while KSL exhibited IC_50_ values of 1.94 µM against MCF-7 cells and 0.99 µM against HT29 human colon cancer cells [30], suggesting that Siye may have strong cytotoxic potential compared with several reported algal lectins, although direct comparison should be interpreted cautiously due to differences in experimental systems. This enhanced activity may be attributed to its optimized glycan-binding interfaces, as suggested by our previous in silico analysis [14]. Moreover, unlike many plant lectins [31], Siye does not agglutinate human erythrocytes, suggesting a potentially lower hemagglutination-related risk; however, further safety evaluation is required.

Importantly, the identification and validation of Siye further demonstrate the value of artificial intelligence-assisted genome mining in marine drug discovery. Compared with traditional extraction and purification strategies, AI-driven approaches enable the rapid screening and prioritization of bioactive candidates from large-scale genomic resources [32]. The integration of in silico prediction and experimental validation, as presented in this study, provides an efficient framework for accelerating the discovery of novel marine-derived therapeutic agents.

### 3.5. Limitations and Future Directions

Although this study extends the known anti-tumor activity spectrum of Siye to colon cancer HCT116 cells and provides additional evidence from morphological observation, apoptosis analysis, and apoptosis-related gene expression profiling in HCC1937 and HCT116 cells, several limitations should be acknowledged. First, the mechanistic evidence remains preliminary because the current analysis was mainly based on cell morphology, flow cytometry, and mRNA expression. Protein-level and functional validation, such as Western blot analysis of cleaved caspase-3, cleaved caspase-8, cleaved caspase-9, Bax translocation, cleaved PARP, caspase activity assays, and pathway inhibition assays, will be required to confirm the specific apoptotic pathways involved. Second, apoptosis and qRT-PCR assays were performed only at the IC_50_ concentration and at a single time point of 24 h, which limits the evaluation of dose- and time-dependent responses. Third, although mannan co-treatment attenuated Siye-induced morphological changes, the present study did not include quantitative cell viability recovery assays to determine whether mannan can prevent or reduce Siye-induced cytotoxicity. Further studies may include appropriate mannan-only controls and CCK-8-based glycan-competition viability rescue assays to determine whether the cytotoxic effect of Siye is glycan-dependent. Fourth, because the HCC1937 IC_50_ value was derived from previously reported data and the cytotoxicity assays were not performed under fully identical conditions, direct quantitative comparison of IC_50_ values between HCC1937 and HCT116 cells should be interpreted cautiously. Finally, in our previous publication, we performed a preliminary evaluation of the effects of Siye on normal cells, including human umbilical vein endothelial cells (HUVECs) and human erythrocytes [14]. In that study, Siye showed cytotoxicity toward HUVECs, with an IC_50_ value of 0.7534 μg/mL (=0.0278 μM), and did not cause agglutination of human erythrocytes of A, B, O, or AB blood types. However, non-tumorigenic cell lines and in vivo models were not included in the present study; therefore, the selectivity and safety profile of Siye require further evaluation. In particular, future studies should include appropriate normal human cell lines tested under the same experimental conditions as cancer cells, allowing calculation of the selectivity index to quantitatively assess cancer cell-selective cytotoxicity. Future studies should include standardized side-by-side cytotoxicity assays using both cancer and non-tumorigenic cell models, calculation of selectivity indices, multiple concentrations and time points, protein-level validation, and CCK-8-based glycan-competition viability rescue assays with appropriate mannan-only controls to further clarify the glycan-associated cellular effects, cancer cell selectivity, safety profile, and anti-tumor potential of Siye. Future studies may further incorporate advanced AI approaches, such as deep learning-based structure–function prediction and sequence optimization, to enhance the discovery and functional characterization of marine lectins.

### 3.6. Conclusions

In summary, this study demonstrates that the algal lectin Siye exerts significant cytotoxic effects against breast cancer HCC1937 and colon cancer HCT116 cells and promotes apoptosis at the IC_50_ concentration. The observed changes in apoptosis-related gene expression preliminarily suggest that caspase-associated pathways may be involved in the anti-tumor effects of Siye, although further protein-level validation is required. The differential sensitivity between cell lines may be related to their surface glycan profiles and p53 status. Our findings, together with previous reports, position Siye as a promising lead compound for the development of marine-derived anti-tumor therapeutics.

## 4. Materials and Methods

### 4.1. AI-Assisted Identification of Siye

The lectin Siye was identified from the genome of the red alga *Kappaphycus alvarezii* using an artificial intelligence-assisted genome mining strategy, as previously described [14]. Briefly, protein sequences were screened using homology-based approaches combined with machine learning-assisted functional annotation to identify candidates belonging to the *Oscillatoria agardhii* agglutinin homolog (OAAH) family. Structural prediction tools were applied to evaluate the folding patterns and conserved carbohydrate-binding domains of candidate proteins. In addition, computational analyses were performed to predict glycan-binding specificity, allowing prioritization of lectin candidates with potential biological activity. Based on these integrated in silico analyses, Siye was selected for experimental validation in this study.

### 4.2. Cell Lines and Cell Culture

Human breast cancer HCC1937 cells and human colon cancer HCT116 cells, provided by Professor Hongbo Wang’s laboratory, were used in this study. HCC1937 cells were cultured in Roswell Park Memorial Institute Medium 1640 (RPMI-1640; Procell, Wuhan, China) supplemented with 10% fetal bovine serum (FBS; Procell, Wuhan, China), 1% double antibiotic (10,000 U penicillin and 10 mg streptomycin solution; Procell, Wuhan, China), and HCT116 cells in Dulbecco’s Modified Eagle Medium (DMEM; Procell, Wuhan, China) containing 10% FBS, 1% double antibiotic. Both cells were inoculated in T-25 culture flasks and cultured in an incubator at 37 °C in a humid environment containing 5% CO_2_. The culture medium was changed every 2 days, and the cells were passaged every 3 days at a ratio of 1:2~1:4. In total, 90% of the fused cells were digested with trypsin (0.025% pancreatic enzyme) for passaging culture.

### 4.3. Cell Viability Assay

For the survival rate measurement, the collected cells were suspended and their density was adjusted. In 96-well plates, HCC1937 cells were seeded at 6000 cells per well, while HCT116 cells were seeded at 8000 cells per well (both in 100 μL of culture medium); these densities were pre-optimized to ensure optimal cell coverage and exponential growth during the assay. The culture plates were incubated at 37 °C, 5% CO_2_ for 24 h to allow cell attachment. Then, the old culture medium was discarded, and 5-fold diluted 20 μL of lectin Siye (prepared in phosphate-buffered saline (PBS, pH 7.4, 10 mM, Procell, Wuhan, China) and filtered through a 0.22 μm membrane) was added to treat the cells, with final concentration ranges from 100 μg/mL to 0.00128 μg/mL and 80 μL of fresh culture medium, including control wells (containing only cells) and blank wells (only PBS). Each concentration was tested in six replicate wells, and the experiment was performed three times independently. After 24 h, the old culture medium was discarded, and fresh medium containing 10 μL of CCK-8 (Procell, Wuhan, China) solution was added to each well, and the OD value of each well was obtained by measuring the absorbance at 450 nm by the enzyme detection instrument SpectraMax iD5e (Molecular devices, San Jose, CA, USA) after 1 h of incubation, using 600 nm as the reference wavelength. The obtained OD values were substituted into the formula:

$$\mathrm{Cell}\;\mathrm{survival}\;\mathrm{rate}\;=\frac{{\mathrm{OD}}_{\mathrm{sample}}\;-{\;\mathrm{OD}}_{\mathrm{blank}}}{{\mathrm{OD}}_{\mathrm{control}}\;-{\;\mathrm{OD}}_{\mathrm{blank}}}\times \;100\%$$

The calculated cell viability values were imported into the software GraphPad Prism 9.5 software (GraphPad Software, Boston, MA, USA) for graphing to obtain IC_50_ values.

### 4.4. Mannan Inhibits Lectin-Induced Cell Shape Changes

To investigate whether Siye-induced morphological changes are associated with cell surface glycan binding, HCC1937 and HCT116 cells were treated with Siye alone or Siye pre-incubated with mannan. Mannan (M11200, ≥98%) was purchased from Shanghai Acmec Biochemical Co., Ltd. (Shanghai, China). Siye (500 μg/mL) and mannan (2000 μg/mL) were mixed at a 1:5 volume ratio and pre-incubated at room temperature for 1 h prior to use.

Cells were seeded into 6-well plates at a density of 1 × 10^5^ cells/well in 2 mL of complete culture medium and incubated for 24 h to allow attachment. After attachment, equal volumes of the corresponding treatment solutions were added to each well. The control group received an equal volume of PBS. Cell morphology was observed and recorded at regular intervals using an inverted microscope, and representative images obtained at 4 h were used for analysis.

### 4.5. Apoptosis Assay

The apoptosis and necrosis of cells were detected by double staining with Annexin V-FITC/PI Apoptosis Detection Kit (Solarbio, Beijing, China). Cells in good growth condition were collected, prepared as single-cell suspensions, seeded into 6-well plates at a density of 1.8 × 10^5^ cells/2 mL per well, and placed in the incubator at 37 °C with 5% CO_2_ for 24 h. The experimental group was administered the drug according to the IC_50_ value after the cells adhered to the bottom, and control wells and single-staining compensation controls were treated with an equal volume of PBS. The cells were incubated for 24 h after Siye addition, the culture medium was aspirated into a centrifuge tube, and trypsin without EDTA was added to digest the cells and collected. After centrifugation and discarding the supernatant, the cells were washed with 1 mL of precooled PBS at 4 °C, and the cell precipitate was resuspended by adding binding buffer and adjusting the concentration of 1–5 × 10^6^/mL. Then, using the Annexin V-FITC/PI Apoptosis Detection Kit, 5 μL of Annexin V/FITC was added to the control group and the experimental group and incubated for 5 min away from light, and then 5 μL of Propidium Iodide (PI) was added for double staining. Annexin V/FITC and PI were added to the single-positive group for single staining, respectively. After the reaction was completed, apoptosis was analyzed using a BD FACSAria™ flow cytometer (BD Biosciences, San Jose, CA, USA).

### 4.6. mRNA Extraction and qRT-PCR

Total RNA extraction: Total RNA was extracted using the SteadyPure Quick RNA Extraction Kit (Accurate Biotechnology, Changsha, China). The culture medium from the cultured cells was discarded after the addition of Siye. Cells were washed with PBS buffer. After discarding the PBS buffer, 500 μL of Buffer QLS lysate was added to the cells for lysis. The homogenate, which was blown until it was clear and not sticking to the silk, was transferred to a centrifuge tube and allowed to stand for 2 min at room temperature. After 2 min, an equal volume of 100% ethanol was added and mixed well. The mixture was subjected to RNA adsorption using Quick RNA Mini Column, and 700 μL of Buffer QWB was added for purification; the purified RNA was eluted by adding 200 μL of RNase Free Water. Then, the purity and concentration of RNA were measured by ultra-micro spectrophotometer NanoDrop 2000 (Thermo Fisher Scientific, Waltham, MA, USA).

RNA reverse transcription of Complementary DNA (cDNA): Extracted total RNA was reverse transcribed into cDNA using the Evo M-MLV Reverse Transcription Premix Kit (Accurate Biotechnology). Genomic DNA (gDNA) was removed using a system containing 2 μL of gDNA Clean Reaction Mix Ver.2, 7 μL of Total RNA, and 1 μL of RNase-free water for 2 min at 42 °C. To the gDNA-removed reaction solution, 4 μL of 5× Evo M-MLV RT Reaction Ver.2 and 6 μL of RNase-free water were added for 15 min at 37 °C, and reverse transcription was performed for 5 s at 85 °C to obtain the cDNA. The reverse transcription was carried out according to the instructions of the kit, and all reaction solutions were prepared at 4 °C. After reverse transcription, the cDNA was stored at −20 °C in a refrigerator.

qPCR: The concentration of cDNA obtained from reverse transcription was diluted to 40 ng/μL. The diluted cDNA was subjected to real-time quantitative gene amplification fluorescence detection using SYBR^®^ Green Pro Taq HS Premix qPCR kit (Accurate Biotechnology). The reaction system was 20 µL, containing 10 µL 2× SYBR Green Pro Taq HS Premix, 0.4 μL Primer-F (10 p), 0.4 µL Primer-R (10 p), 2 µL cDNA, and 7.2 µL RNase-free water. The qPCR system was added to a 96-well plate, sealed, and centrifuged at 12,000 rpm for 1 min to remove air bubbles. Transfer the 96-well plate to the qPCR instrument, set the relevant program parameters, and start the program. Program parameters were: 95 °C, 6 min; 95 °C, 15 s; 55 °C, 60 s; 40 cycles. *GAPDH* was used as the internal reference gene, and finally, the gene expression was calculated using the 2^−ΔΔCT^ method [33]. The primer sequences were as follows: *Bax* forward, 5′-CGGGTT-GTCGCCCTTTTCTA-3′, *Bax* reverse, 5′-AAGATGGTCACGGTCCAACC-3′; *Bcl*-2 forward, 5‘-GACTGAGTACCTGAACCGGC-3′, *Bcl*-2 reverse, 5′-GTTGACTTCAC-TTGTGGCCC-3′; *Casp3* forward, 5‘-CATGGAAGCGAATCAATGGACT-3′, *Casp3* reverse, 5′-CTGTACCAGACCGAGATGTCA-3′; *Casp8* forward, 5‘-CAGAA-GAGCCAGGGTGGTTA-3′, *Casp8* reverse, 5′-GGGTTCTTGCTTCCTTTGCG-3′, *Casp9* forward, 5‘-CTCAGACCAGAGAGATTCGCAAAC-3′, *Casp9* reverse, 5′-GCATTTCCCCTCAAACTCTCAA-3′; *TP53* forward, 5′-GAGGTT-GGGCTCTCTGACTGTACC-3′, *TP53* reverse, 5′-TCCGTCCCAGTAGATTACCAC-3′; *GAPDH* forward, 5‘-GACAGTCAGCCGCATCTTCT-3′, *GAPDH* reverse, 5′-TTAAAAGCAGCCCTGGTGAC-3′.

### 4.7. Statistical Analyses

All experiments were performed at least three times independently. Data are presented as mean ± SEM. IC_50_ values were calculated by nonlinear regression using the log(inhibitor) vs. normalized response–variable slope model. Before group comparisons, data distribution was assessed using the Shapiro–Wilk normality test. Therefore, gene expression comparisons between the control and Siye-treated groups were performed using an unpaired two-tailed Student’s *t*-test. Each gene was analyzed independently. *p* < 0.05 was considered statistically significant. Analyses were conducted using GraphPad Prism 9.5 software (GraphPad Software, Boston, MA, USA).

## Data Availability

The original data are available from the corresponding author on request.
